# Carboxyphenolate Coordination Frameworks for High‐Voltage Calcium Storage

**DOI:** 10.1002/advs.75481

**Published:** 2026-05-11

**Authors:** Vasudeva Rao Bakuru, Darsi Rambabu, Xiaodong Lin, Robert Markowski, Petru Apostol, Viliam Frano, Xiaolong Guo, Taniya Purkait, Shubhadeep Pal, Tom Goossens, Da Tie, Augustin Ramackers, Alexandru Vlad

**Affiliations:** ^1^ Institute of Condensed Matter and Nanosciences Molecular Chemistry Materials and Catalysis Université catholique de Louvain Louvain la Neuve Belgium; ^2^ Department of Physics School of Advanced Science VIT‐AP University Amaravati Andhra Pradesh India; ^3^ WEL Research Institute Wavre Belgium

**Keywords:** calcium‐ion storage, coordination framework, divalent cation electrodes, high‐voltage, organic positive electrode

## Abstract

Calcium batteries are attractive candidates for next‐generation energy storage offering both their high theoretical energy density and the advantage of abundant, naturally available calcium. However, the design of high‐voltage positive electrodes for Ca‐ion‐containing systems remains challenging, with only few examples reported to date. The main difficulties arise from the synthesis and ion‐storage characteristics of Ca‐based materials, as the high polarizing power of Ca^2+^ limits diffusion. Accommodating Ca^2+^ requires flexible or disordered frameworks that facilitate calcium‐ion mobility. Herein, we investigate conjugated carboxyphenolate coordination frameworks (Ca_2_ **‐*M*‐**THBPD; M = Mg^2+^, Ca^2+^, Ba^2+^; wherein THBPD = 2,2′,5,5′‐tetraoxido‐[1,1′‐biphenyl]‐4,4′‐dicarboxylate) as amorphous organic positive electrode materials. Ca_2_‐***M***‐THBPD operates above 3.5 V vs. Ca^2+^/Ca (median discharge voltage 3.55 V) with low hysteresis and polarization, enabled by the synergy of amorphous disorder, enolate‐quinone redox activity, and inductive spectator‐cation effects. The electrode delivers a discharge capacity of 120 mAh g^−1^ with a Coulombic efficiency of 99.8%, retaining 75% of its initial capacity after 200 cycles at a C/20 rate. This study demonstrates, the use of reduced‐state conjugated carboxyphenolate frameworks as active materials for high voltage divalent cation storage, highlighting how spectator cations and framework flexibility influence redox potential and long‐term stability in Ca‐ion batteries.

## Graphical abstract

A hexaanionic carboxyphenolate coordination framework, Ca_2_‐***M***‐THBPD (M = Mg, Ca, Ba), exhibits ligand‐centered enolate‐quinonoid redox activity enabling tunable, high‐voltage Ca^2^^+^ storage (3.5 V vs Ca^2+^/Ca) modulated by spectator cations. The amorphous structure accommodates reversible divalent‐ion insertion, contributing to enhanced cycling stability.

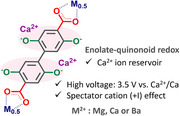

## 1 Introduction

Calcium batteries (CaBs) are emerging as a promising alternative to lithium‐ion technology for large‐scale energy storage, offering advantages in cost, resource availability, and sustainability [1, 2, 3, 4]. However, their development remains nascent due to challenges in electrode design and sluggish ion transport [1, 2, 3, 4]. The divalent nature of Ca^2+^ enables high theoretical volumetric energy density and competitive gravimetric capacity, while the abundance and low cost of calcium make this technology economically attractive and scalable for practical applications [5]. Nevertheless, the strong electrostatic interactions between Ca^2+^ and host lattices hinder ionic diffusion, in contrast to the faster transport observed with monovalent Li^+^ or Na^+^ ions [2, 6, 7]. The relatively large ionic radius of Ca^2+^ further demands host structures with wide diffusion channels to enable efficient intercalation and reversible redox processes. Collectively, these characteristics impose critical constraints on the design of high‐performance Ca‐storage electrode.

**SCHEME 1:**
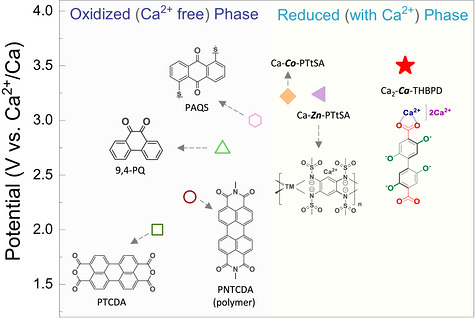
Representative redox‐active organic compounds and coordination polymers reported for calcium‐ion storage. The oxidized (Ca^2+^−free) and reduced (Ca^2+^−coordinated) phases are shown on the left and right, respectively.

Efforts to develop high‐voltage Ca‐storage electrodes have primarily focused on inorganic positive electrode chemistries (Table S2) [8, 9, 10, 11, 12, 13, 14, 15]. Representative examples include layered chalcogenides and oxides such as TiS_2_, [16] V_2_O_5_, [17] α‐MoO_3_, [18] as well as spinel‐type transition‐metal oxides (e.g., Ca_x_Mn_2_O_4_), [19] and Prussian blue analogues (e.g., MnFe(CN)_6_) [20]. Although these materials support reversible Ca^2+^ insertion, they exhibit limited stability (<100 cycles) and poor rate performance due to sluggish Ca^2+^ diffusion and lattice strain‐induced structural degradation [20, 21]. Developing high‐voltage, durable inorganic calcium positive electrodes therefore remains a major challenge, motivating the exploration of alternative chemistries. Organic redox‐active molecules offer a promising route, featuring tunable redox potentials, structural flexibility, and lower environmental impact [22]. Their modular design enables precise control of redox behavior across Li‐ion and multivalent systems [6, 23, 24]. However, only a limited number of organic materials have shown satisfactory Ca‐ion storage, while being also synthesized and assembled in their pristine oxidized states (Scheme 1, Table S2) which remains unsuitable for practical application. For example, perylene‐3,4,9,10‐tetracarboxylic dianhydride (PTCDA) [25] and poly‐1,4,5,8‐naphthalenetetracarboxylic diimide (PNTCDA) [26] operate at relatively lower potentials of 2.3 and 2.0 V vs. Ca/Ca^2+^, respectively. Phenanthrenequinone (PQ) reversibly stores Ca^2+^ via its C═O/C─O redox at average voltage of 2.75 V, aided by enolization and structural flexibility [27]. In contrast, poly(anthraquinonyl sulfide) ‐ carbon nanotubes (PAQS‐CNT) composites [28] were found to fade rapidly due to surface passivation on Ca metal anode [29]. Anhydride‐based compounds (pyromellitic dianhydride (PMDA), 1,4,5,8‐naphthalenetetracarboxylic dianhydride (NTCDA), PTCDA) show decent initial capacities but rapid decay, reflecting intrinsic Ca^2+^ cycling challenges [30]. Recently, amorphous coordination frameworks, exemplified by Ca‐Zn‐PTtSA (PTtSA^4−^, benzene‐1,2,4,5‐tetra‐methylsulfonamide), have been demonstrated as positive electrodes for CaBs, operating at 3.2 V vs. Ca^2+^/Ca with 99% capacity retention over extended cycling [31]. This work revealed that, unlike crystalline hosts, amorphous frameworks leverage structural disorder and electronic delocalization, which facilitate Ca^2+^ insertion and extraction while minimizing strain and enhancing ion mobility [32].

Herein, we report amorphous hexa‐anionic conjugated carboxyphenolate frameworks, Ca_2_‐***M***‐THBPD (M = Mg^2+^, Ca^2+^, or Ba^2+^; THBPD = 2,2′,5,5′‐tetraoxido‐[1,1′‐biphenyl]‐4,4′‐dicarboxylate) for high voltage and efficient Ca‐ion storage. The materials are synthesized through direct proton exchange method involving sodium methoxide to yield the sodium form, followed by cation exchange to incorporate the corresponding divalent metal ions. In these materials, the spectator cations (Mg^2+^, Ca^2+^, or Ba^2+^) coordinate to carboxylate groups, whereas two Ca^2+^ ions serve as charge compensators by coordinating to four phenolate sites. The Ca_2_‐***M***‐THBPD positive electrode operates at 3.5 V vs. Ca^2+^/Ca, with the high potential enabled by electronic modulation through the spectator ion effect. The amorphous nature of the frameworks facilitates Ca^2+^ extraction/storage, and the ligand core provides a reversible enolate‐quinone redox couple for calcium‐ion storage. The electrode exhibits a discharge capacity of 120 mAh g^−1^ with a Coulombic efficiency of 99.8% at a C/20 rate. These results establish the first conjugated carboxyphenolate frameworks for reversible calcium‐ion storage, providing a platform for high‐voltage, durable divalent metal‐ion batteries.

## 2 Results and Discussion

**FIGURE 1:**
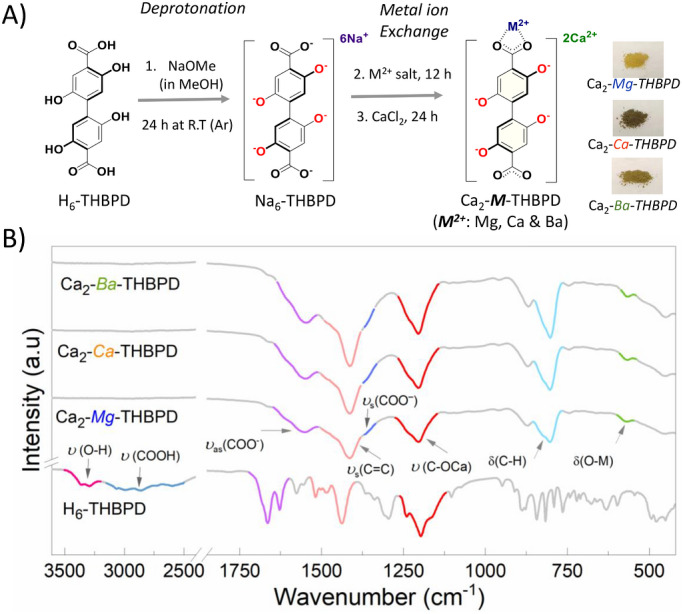
(A) Schematic illustration of the synthesis process of Ca_2_‐***M**‐*THBPD (M = Mg^2+^, Ca^2+^, & Ba^2+^) from H_6_‐THBPD. (B) FTIR spectra comparing H_6_‐THBPD with Ca_2_‐***M**‐*THBPD phases, highlighting shifts in key vibrational bands upon alkali metal incorporation.

The H_6_‐THBPD redox linker contains two hydrobenzoquinone groups capable of multi‐electron transfer involving up to four electrons, facilitating the (de)insertion of divalent cations through enolate‐quinone redox transitions. The two carboxylate groups of THBPD^6−^ stabilize the framework by coordinating spectator cation, which additionally increases the redox potential through the inductive (+I) effect [33]. This coordination also promotes the formation of polymeric frameworks that enhance storage performance by limiting solubility. Typically, Ca_2_‐***M***‐THBPD (M^2+^ = Mg, Ca, Ba) was synthesized in a one‐pot process via sequential addition of cation species precursors. The H_6_‐THBPD ligand was first deprotonated with sodium methoxide to yield the sodiated hexaanionic form, followed by stepwise cation exchange. For Ca_2_‐***M***‐THBPD (M = Mg^2+^ and Ba^2+^), two Na^+^ ions and the remaining four sodium ions were successively replaced by one spectator M^2+^ ion and two equivalents of Ca^2+^ storage cations, respectively. In contrast, for M^2+^ = Ca, the sodium exchange occurred in a single addition step to afford Ca_2_‐***Ca***‐THBPD (Figure 1A). Fourier transform infrared (FTIR) spectroscopy was employed to confirm coordination between the cation and the ligand, particularly through the carboxylate (─COO^−^) and phenolate/enolate (─C─O^−^) groups. The disappearance of the O─H stretching bands corresponding to phenolic (3250–3420 cm^−1^) and carboxylic acid (3100–2700 cm^−1^) groups, together with the red shift of the carbonyl stretching band (ν_C═O_) from 1663 cm^−1^ to 1555, 1545, and 1537 cm^−1^ for Ca_2_ **‐*Mg*‐**THBPD, Ca_2_ **‐*Ca*‐**THBPD, and Ca_2_ **‐*Ba*‐**THBPD, respectively, provides clear evidence of ligand deprotonation and subsequent metal coordination (Figure 1B). Additionally, the phenolate ν_C‐O_/δ_C─O─H_ band shifted from 1195 to 1205 cm^−1^ in all three materials, further supporting proton exchange. Powder x‐ray diffraction revealed that all Ca_2_‐***M***‐THBPD compounds are amorphous (Figure S1A) which might be attributed to the strongly basic, kinetically controlled synthesis conditions, as well as the flexible THBPD ligand, mixed divalent cations, and large Ca^2+^ ions that collectively hinder ordered crystallization and efficient packing. The specific surface areas, determined by N_2_ adsorption at 77 K are 167, 96, and 46 m^2^ g^−1^ for Ca_2_‐***Mg***‐THBPD, Ca_2_‐***Ca***‐THBPD, and Ca_2_‐***Ba***‐THBPD, respectively, indicating limited porosity and supporting the formation of compact, low‐order frameworks Figure S1B,C. Scanning Electron Microscopy (SEM) reveals distinct morphologies with particle sizes of approximately 100 nm (Figure S1D–F): Ca_2_‐***Mg***‐THBPD forms dense, compact aggregates; Ca_2_‐***Ca***‐THBPD displays loosely connected aggregates; and Ca_2_‐***Ba***‐THBPD exhibits hierarchical, interconnected networks. While this disordered structure precludes precise structural determination, it is advantageous for Ca^2+^ storage, as it accommodates ion extraction/insertion with reduced mechanical strain and enhanced ion mobility. Thermogravimetric analysis (TGA) reveals that all three Ca_2_‐***M***‐THBPD positive electrode materials exhibit thermal stability up to approximately 300°C (Figure S2). Elemental (CH) and inductively coupled plasma optical emission spectroscopy (ICP‐OES) analyses confirmed the compositions of all studied phases (Table S1).

### 2.1 Electrochemical Calcium Storage in Ca_2_‐*M*‐THBPD Positive Electrode Materials

**FIGURE 2:**
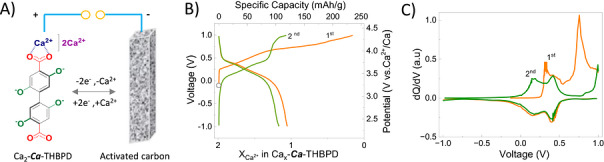
(A) Schematic representation of the cell configuration used to study Ca‐ion storage in Ca_2_‐***Ca***‐THBPD positive electrode and an activated carbon (AC) counter electrode. (B) First two charge–discharge cycles recorded at 0.1 C between −1 and 1 V in cells with activated carbon counter electrode in 1 m Ca(TFSI)_2_/acetonitrile electrolyte, along with the corresponding potential (V) profile vs. Ca^2+^/Ca. (C) Differential capacity (dQ/dV) plot for the first two cycles of Ca_2_‐***Ca***‐THBPD, highlighting the evolution of redox processes.

Galvanostatic charge–discharge (GCD) studies evaluated the ability of Ca_2_‐***M***‐THBPD positive electrodes to reversibly release and store Ca^2+^ under ambient conditions. To limit the blocking nature and parasitic reactions from Ca metal [34, 35], half‐cells used activated carbon (AC) as both counter electrode (in large capacitance excess), providing a stable platform to probe intrinsic electrochemical behavior [36]. Due to its capacitive (de)adsorption properties, AC enables reliable assessment of cation (de)insertion. Electrochemical testing of Ca_2_‐***Ca***‐THBPD positive electrode began with charging, corresponding to Ca^2+^ ions extraction (Figure 2A,B; Figure S5B) at rate of C/10 initial charge shows a sloped profile from −1.0 to 1.0 V vs. AC, corresponding to 1.84 Ca^2+^ extraction and delivering 235 mAh g^−1^ (theoretical: 255 mAh g^−1^). The GCD discharge profile exhibits a broad plateau, indicating partial reversibility, likely limited by structural rearrangements. The second cycle shows a more symmetric profile, reflecting electrode‐electrolyte interface stabilization and improved redox kinetics. The positive electrode potential, determined using a three‐electrode cell (Figure S4), shows that Ca_2_‐***Ca***‐THBPD delivers a median discharge voltage of 3.55 V vs. Ca^2+^/Ca at 50 % capacity, slightly above the average discharge voltage of 3.5 V, reflecting a marginally higher practical energy output. To the best of our knowledge, this represents the highest reported value for calcium‐storage positive electrodes (Table S2). This high operating voltage is attributed to the fully calcium‐based framework stabilized exclusively by Ca^2+^‐ligand interactions, which enables an elevated redox potential through its specific chemical design and associated charge‐storage mechanism. The corresponding dQ/dV profiles (Figure 2C; Figure S5B) reveal a clear evolution in redox behavior upon cycling. In the first cycle, broad and defined peaks (0.3 and 0.75 V vs. AC) during charge and discharge indicate possible framework structural re‐arrangement associated to ligand oxidation as well as 2 Ca^2+^ extraction. By the second cycle, two distinct oxidation peaks appear at 0.2 and 0.4 V, corresponding to Ca^2+^ extraction and framework oxidation, accompanied by two sharper reduction peaks (0.18 and 0.38 V), indicative of more defined Ca^2+^ uptake. This evolution reflects electrochemical activation and the establishment of reversible redox processes within Ca_2_‐***Ca***‐THBPD. During extended cycling, gradual capacity fade was observed, likely associated with degradation or dissolution of the oxidized (quinone) form of Ca_2_‐***Ca***‐THBPD in the electrolyte (Figures S3 and S5) [37]. Such processes contribute to the poor cycling stability observed under full calcium extraction. This was further revelaed by SEM image of post‐cycled Ca_2_‐***Ca***‐THBPD shows slight surface smoothing and rounding, likely arising from partial dissolution or degradation of the oxidized phase and subsequent interfacial reorganization (Figure S6), This observation was corroborated by UV–vis spectroscopy after full charge, which revealed a 330 nm peak corresponding to the quinone moiety in the oxidized phase (Figure S5E) [37]. In this regard, two factors may account for this behavior: (i) intrinsic instability of the oxidized phase at high potential or (ii) dissolution of the pristine or oxidized species leading to active material loss. Given that the fully oxidized four‐electron phase is experimentally stable and isolable, the latter is considered the dominant cause.

**FIGURE 3:**
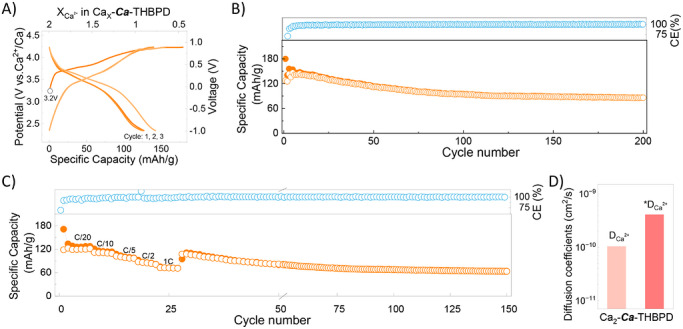
(A) Galvanostatic charge–discharge profiles of the Ca_2_‐***Ca***‐THBPD positive electrode material over the first three cycles at a rate of C/20, measured between −1.0 and 0.9 V vs. activated carbon in 1 M Ca(TFSI)_2_/acetonitrile electrolyte. (B) Long‐term cycling stability at rate of C/20. (C) Different capacity‐rate test performance (C/20 to 1C) of the Ca_2_‐***Ca***‐THBPD positive electrode. (D) Evaluated diffusion coefficients for the high‐potential region (^*^D) and low‐potential region(D) from GITT.

To evaluate long‐term cyclability, the three positive electrode materials: Ca_2_‐***Mg***‐THBPD, Ca_2_‐***Ca***‐THBPD, and Ca_2_‐***Ba***‐THBPD were tested after blending with Ketjen Black (KB600), chosen as the conductive additive to suppress dissolution, enhance cycling stability and rate performance. A consistent open‐circuit voltage (OCV) of approximately 3.25 V vs. Ca^2+^/Ca was observed for all materials (Figure 3A), indicative of a common redox‐active ligand environment within the Ca_2_‐***M***‐THBPD positive electrodes. Figure 3 summarizes the electrochemical performance of Ca_2_‐***Ca***‐THBPD positive electrode, taken as the representative electrode. The initial galvanostatic charge–discharge profiles (Figure 3A; Figure S5F), long‐term cycling stability (Figure 3B), and rate capability (Figure 3C) collectively confirm the materials stable performance. The Ca_2_‐***Ca***‐THBPD positive electrode exhibits sloped voltage profiles during the first three cycles, consistent with a solid‐solution reaction mechanism. At a C/20 rate, it delivers one Ca^2+^ capacity (120 mAh g^−1^) ion per formula unit out of the theoretical two Ca^2+^ capacity (255 mAh g^−1^). A minor capacity contribution of 30 mAh g^−1^ originates from the conductive additive (KB600, Figure S9). In comparison, Ca_2_‐***Mg***‐THBPD and Ca_2_‐***Ba***‐THBPD deliver capacities of 85 mAh g^−1^ (0.6 Ca^2+^) and 130 mAh g^−1^ (1.25 Ca^2+^), respectively (Figures S10 and S12). The systematic increase in capacity with the ionic radius of the framework cation (from Mg^2+^ to Ba^2+^) arises from lattice expansion that enhances Ca^2+^ diffusion and ion accessibility [38]. Th suggest that larger metal nodes impart greater framework flexibility and enlarge the diffusion channels, thereby facilitating more efficient ion transport within the structure [39]. During cycling, the Ca_2_‐***Ca***‐THBPD positive electrode exhibits progressive stabilization of both capacity and Coulombic efficiency over the initial three cycles, reaching a reversible capacity of 120 mAh g^−1^ with minimal polarization. Following this activation period, the electrode retains over 75% of its initial capacity after 200 cycles, maintaining an average Coulombic efficiency of above 99.8%, indicative of highly reversible Ca^2+^ extraction and uptake. Ca_2_‐***Ca***‐THBPD is a fully calcium‐based framework stabilized exclusively by Ca^2+^‐ligand interactions, a spectator‐cation inductive effect and enables Ca^2+^‐storage via its amorphous nature. Comparable stability is observed for Ca_2_‐***Mg***‐THBPD and Ca_2_‐***Ba***‐THBPD electrodes (Figures S11 and S13), which sustain Coulombic efficiencies exceeding 98.5% and 99.6%, respectively, over 100 cycles. Rate capability tests from C/20 to 1C (Figure 3C) further highlight the robustness of Ca_2_‐***Ca***‐THBPD positive electrode, delivering 60 mAh g^−1^ (0.5 Ca^2+^) at 1C with stable coulombic efficiency. The favorable rate capability is attributed to efficient Ca^2+^ diffusion within the framework. Diffusion coefficients(D) determined from the galvanostatic intermittent titration technique (GITT) range from 4.1 × 10^−10^ (^*^D at high‐potential) to 1.0 × 10^−10^ cm^2^ s^−1^ (D at low‐potential) (Figure 3D; Figure S7), indicating relatively fast multivalent ion transport in the solid‐state electrode. Nyquist and Bode analyses (Figure S8A–D) further reveal similar ohmic resistance, a mid‐frequency depressed semicircle with phase angles around 45° corresponding to charge‐transfer processes, and a low‐frequency inclined line characteristic of Ca^2+^ diffusion, demonstrating that the electrode kinetics are governed by structure‐dependent ion transport and interfacial charge transfer [31, 40, 41]. This behavior likely arises from the open‐framework architecture and redox‐active ligand environment, which together facilitate reversible Ca^2+^ insertion and extraction while limiting kinetic constraints.

### 2.2 Redox Mechanism of Ca_2_‐*M*‐THBPD Positive Electrode

**FIGURE 4:**
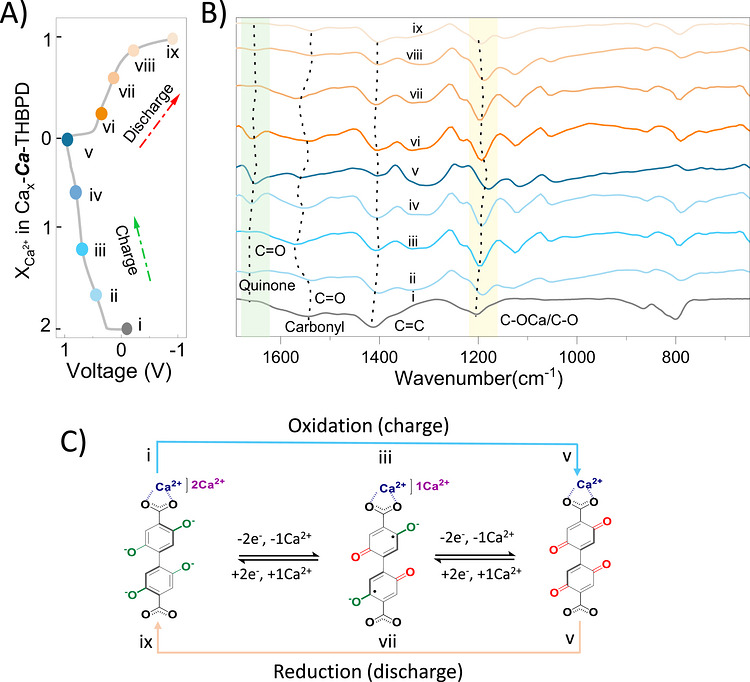
(A) Voltage‐composition profile of the Ca_2_‐***Ca***‐THBPD positive electrode cycled at a 0.1C rate, illustrating the reversible redox behavior associated with Ca^2+^ release and uptake. (B) Ex situ infrared (IR) spectra of the electrode composite acquired at selected states during cycling; pristine (i), charged states (ii–v), and discharged states (vi–ix). Spectral evolution correlates with the progressive oxidation and reduction of redox‐active functional groups, as indicated by characteristic vibrational modes at each stage corresponding to the points marked in (A). (C) Proposed charge–discharge intermediates via quinone/phenolate redox in Ca_2_‐***Ca***‐THBPD positive electrode.

To gain further insight into the redox mechanism during electrochemical cycling of the Ca_2_‐***Ca***‐THBPD phase, Ex situ FTIR spectroscopy was performed on electrode materials collected at different states of charge (SOC) during the charge and discharge processes (Figure 4A,B). In particular, spectra were recorded at selected electrochemical states: pristine electrode (i), and charged and discharged states {25% SOC (ii, viii), 50% SOC (iii, vii), 75% SOC (iv, vi), and 100% SOC (v, ix)}. The FTIR spectrum of the fully discharged electrode (ix) closely resembles that of the pristine material (i), indicating a high degree of structural reversibility. In contrast, the spectra collected at charged states (ii, iii, iv, and v) display the emergence of a characteristic band at 1654 cm^−1^, corresponding to the quinonoid carbonyl (ν_C═O_) vibration band, accompanied by attenuation of the phenolate ν_C‐O‐Ca_ stretching mode [33, 42, 43]. These spectral variations indicate weakening of metal‐ligand coordination upon oxidation. Further support comes from the chemically oxidized Ca_0_‐***Ca***‐THBPD, which exhibits a distinct carbonyl stretch at 1660 cm^−1^, confirming quinone formation associated with ligand oxidation (Figure S14). Upon subsequent discharge (vi, vii, viii, and ix), the ν_C═O_ band diminishes while the phenolate ν_C‐O‐Ca_ feature reappears, confirming the reversible re‐establishment of the enolate coordination environment. Consistently, the dQ/dV profiles (Figure 2C) display oxidation peaks at approximately 0.3 and 0.75 V during charge, corresponding to Ca^2+^ extraction and quinone formation in Ca_2_‐***Ca***‐THBPD positive electrode, and reduction peaks at 0.38 and 0.18 V during discharge, reflecting the reversible Ca^2+^ uptake and regeneration of the calcium phenolate form after the initial activation, in agreement with the proposed redox mechanism. Collectively, these observations reveal a redox process governed by a reversible enolate‐quinonoid carbonyl transformation is localized on the organic linker. The proposed reaction intermediates associated with this process are illustrated in Figure 4C.

**FIGURE 5:**
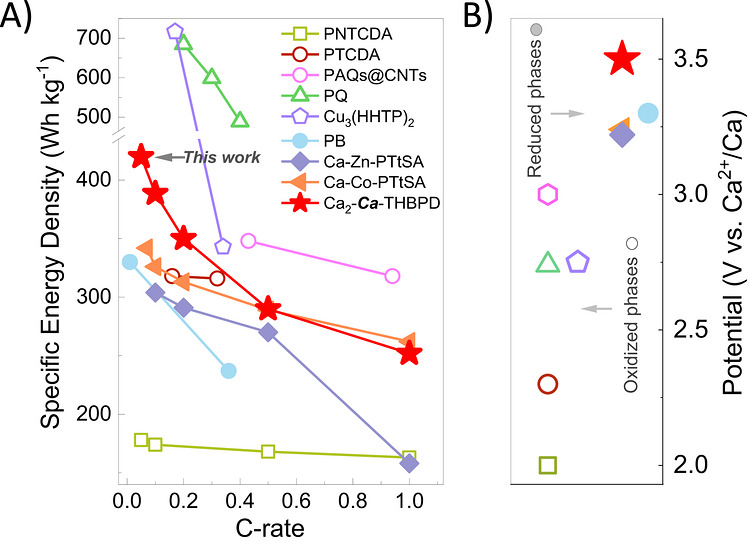
(A) Comparison of energy density vs. C‐rate performance for Ca_2_‐***Ca***‐THBPD (red stars, *this work*) and representative state‐of‐the‐art Ca‐ion positive electrode materials including PNTCDA [26] (light green squares), PTCDA [25] (dark brown circles), PAQs@CNTs [28] (Pink pentagons), PQ (green triangles) [27], Cu_3_(HHTP)_2_ [45], Prussian Blue (PB) [46], Ca‐Zn‐PTtSA [31] (purple diamonds), and Ca‐Co‐PTtSA [31] (orange triangles). (B) Average discharge potentials vs. Ca^2+^/Ca for reduced (closed circle) and oxidized (open circle) phases.

Finally, a comparative analysis of voltage profiles, cycling behavior, and sustainability metrics (Figure 5A; Table S2) highlights the competitive performance of Ca_2_‐***Ca***‐THBPD among reported Ca‐based positive electrodes (oxidized and reduced phases) [25, 26, 27, 44]. Notably, its redox‐active framework sustains one of the highest average voltages (3.5 V vs. Ca^2+^/Ca) and high specific energy density at the material level (Figure 5B) among reported organic Ca^2+^ positive electrodes while maintaining stable cycling and high‐rate capability. This synergy of high voltage, structural robustness, and composition based solely on earth‐abundant elements establishes Ca_2_‐***Ca***‐THBPD as a promising platform for next‐generation calcium‐based energy storage.

## 3 Conclusion

In summary, we developed a family of conjugated hexa‐anionic carboxyphenolate frameworks, Ca_2_‐***M***‐THBPD (M^2+^ = Mg, Ca, Ba), that enable high‐voltage and reversible Ca^2+^ storage. Synthesized in their reduced Ca^2+^‐reservoir form, these frameworks exhibit ligand‐centered redox activity via reversible enolate‐quinonoid transformations, with divalent cations ensuring charge balance and structural stability. The amorphous framework accommodates Ca^2+^ release and uptake with minimal strain, while spectator cations modulate the electronic structure via inductive effects and interactions with the ligand core. The representative Ca_2_‐***Ca***‐THBPD electrode operates above 3.5 V vs. Ca^2+^/Ca (median discharge voltage 3.55 V), maintains stable capacity and rate performance, and preserves structural integrity during cycling. This work introduces conjugated carboxyphenolate frameworks as a promising class of high‐voltage organic positive electrodes, offering molecular‐level design principles for efficient and sustainable divalent‐ion storage.

## 4 Experimental Section/Methods

### 4.1 Chemicals and Reagents

Anhydrous methanol (99.9%, Alfa Aesar), anhydrous diethyl ether (99%, Alfa Aesar), Sodium methoxide (98%, Sigma Aldrich), anhydrous barium (II) chloride (98%, Alfa Aesar), anhydrous Mg(TFSI)_2_ (99.5%, Solvionic), anhydrous calcium bis(trifluoromethanesulfonyl)imide Ca(TFSI)_2_ (99.5%, Solvionic) anhydrous calcium chloride (98%, Sigma Aldrich). Anhydrous solvents were employed within an Ar‐filled glovebox, with O_2_ and H_2_O content maintained below 1 ppm. All chemicals and reagents were used as received, unless specifically mentioned.

Coin cell assembly parts (stainless steel, SS‐316), active carbon, conductive carbon and polytetrafluoroethylene (PTFE) were purchased from TOB New Energy Technology Co., Ltd. (Xiamen, China). The Glass fiber separator (GF/D) was purchased from Whatman. Active carbon fabric (ACC‐5092‐20) was bought from Kynol.

### 4.2 Materials Characterization

X‐ray powder diffraction (XRD) patterns were collected on STOE DARMSTADT Transmission diffractometer system using Mo Kα1 radiation with a wavelength of 0.70930 Å and an operating voltage of 40 Kv. Fourier Transform Infrared (FTIR) spectroscopy was conducted using an Agilent Technologies Cary 630 FTIR operated in ATR mode, covering the wavenumber range from 4000 to 400 cm^−1^ at a resolution of 4 cm^−1^ and 64 scans for data acquisition. Elemental analysis (CH) was performed using the Thermo Scientific FlashSmart Elemental Analyzer. The metal contents were analyzed by inductively coupled plasma atomic emission spectroscopy (ICPAES). Thermogravimetric analysis (TGA) was carried out under N_2_ atmosphere using Mettler Toledo TGA/DSC 3^+^ STARe at a heating rate of 5°C per minute.

### 4.3 Cell Assembly

The electrochemical performance evaluation of Ca_2_‐***M***‐THBPD (M^2+^: Mg, Ca, or Ba) in the solid phase (galvanostatic charge–discharge) typically involved the use of two‐electrode coin cells, which were assembled within an argon‐filled glovebox.

Preparation of Ca_2_‐***M***‐THBPD composite: The positive electrode was prepared by manually grinding 50 wt.% of the active material with 40 wt.% of conductive carbon (Ketjen Black or Super P) and 10 wt.% of PTFE binder. The resulting composite powder was applied onto the positive side of a coin cell case (CR2032, AISI 316L stainless steel) and pressed directly onto the case using a stainless‐steel rod. The active material loading was approximately 2 mg. Two glass fiber separators (19 mm in diameter) were placed on top of the positive electrode, followed by the addition of 75 µL of electrolyte. Two activated carbon (32 mg, AC, 13 mm) cloths, serving as counter electrodes, were then placed on the anode side above the separator, and an additional 75 µL of electrolyte was added (AC capacitance was 32 mAh/g/V at 1 mA/g current density). A stainless steel (316) current collector and spring were placed above the anode, and the cell was sealed with the negative case under a pressure of 0.7 tons. Galvanostatic charge–discharge tests were performed at room temperature using Neware and Biologic battery testing systems. Galvanostatic charge–discharge experiments were conducted at room temperature using a Neware battery testing equipment. For cation storage, a freshly prepared electrolyte consisting of 1 m Ca(TFSI)_2_ in acetonitrile (ACN) or propylene carbonate (PC) was employed. It is worth noting that, given the high operating voltage of these materials, these electrolytes were selected as the most suitable option owing to its high anodic stability.

## Conflicts of Interest

The authors declare no conflicts of interest.

## Supporting information




**Supporting File**: advs75481‐sup‐0001‐SuppMat.docx.

## Data Availability

The data that support the findings of this study are available from the corresponding author upon reasonable request.

## References

[1] A. Ponrouch , C. Frontera , F. Bardé , and M. R. Palacín , “Towards a Calcium‐Based Rechargeable Battery,” Nature Materials 15 (2016): 169–172, 10.1038/nmat4462. 26501412

[2] M. E. Arroyo‐De Dompablo , A. Ponrouch , P. Johansson , and M. R. Palacín , “Achievements, Challenges, and Prospects of Calcium Batteries,” Chemical Reviews 120 (2020): 6331–6357, 10.1021/acs.chemrev.9b00339. 31661250

[3] M. Armand and J.‐M. Tarascon , “Building Better Batteries,” Nature 451 (2008): 652–657, 10.1038/451652a. 18256660

[4] S. Energy , “Time for Lithium‐Ion Alternatives,” Nature Energy 7 (2022): 461.

[5] A. H. Johnstone , “CRC Handbook of Chemistry and Physics—69th Edition Editor in Chief R. C. Weast, CRC Press Inc., Boca Raton, Florida, 1988, pp. 2400, price £57.50. ISBN 0–8493–0369–5,” Journal of Chemical Technology & Biotechnology 50 (1991): 294–295, 10.1002/jctb.280500215.

[6] C. W. Yuan Chen , K. Fan , and Y. Gao , “Challenges and Perspectives of Organic Multivalent Metal‐Ion Batteries,” Advanced Materials 34 (2022): 2200662, 10.1002/adma.202200662. 35364614

[7] I. D. Hosein , “The Promise of Calcium Batteries: Open Perspectives and Fair Comparisons,” ACS Energy Letters 6 (2021): 1560–1565, 10.1021/acsenergylett.1c00593.

[8] M. E. Purbarani , J. Hyoung , and S. T. Hong , “Crystal‐Water‐Free Potassium Vanadium Bronze (K_0.5_V_2_O_5_) as a Cathode Material for Ca‐Ion Batteries,” ACS Applied Energy Materials 4 (2021): 7487–7491, 10.1021/acsaem.1c01158.

[9] C. Zuo , F. Xiong , J. Wang , Y. An , L. Zhang , and Q. An , “MnO_2_ Polymorphs as Cathode Materials for Rechargeable Ca‐Ion Batteries,” Advanced Functional Materials 32 (2022): 2202975, 10.1002/adfm.202202975.

[10] J. Hyoung , J. W. Heo , B. Jeon , and S. T. Hong , “Silver Vanadium Bronze, β‐Ag_0.33_V_2_O_5_: Crystal‐Water‐Free High‐Capacity Cathode Material For Rechargeable Ca‐Ion Batteries,” Journal of Materials Chemistry A 9 (2021): 20776, 10.1039/D1TA03881H.

[11] A. L. Lipson , S. D. Han , S. Kim , et al., “Nickel Hexacyanoferrate, A Versatile Intercalation Host for Divalent Ions From Nonaqueous Electrolytes,” Journal of Power Sources 325 (2016): 646–652, 10.1016/j.jpowsour.2016.06.019.

[12] Z. L. Xu , J. Park , J. Wang , et al., “A New High‐Voltage Calcium Intercalation Host for Ultra‐Stable and High‐Power Calcium Rechargeable Batteries,” Nature Communications 12 (2021): 3369. 10.1038/s41467-021-23703-x PMC8184813 34099694

[13] J. Wang , J. Wang , Y. Jiang , et al., “CaV_6_O_16_·2.8H_2_O with Ca^2+^ Pillar and Water Lubrication as a High‐Rate and Long‐Life Cathode Material for Ca‐Ion Batteries,” Advanced Functional Materials 32 (2022): 2113030, 10.1002/adfm.202113030.

[14] Z. Li , B. P. Vinayan , P. Jankowski , et al., “Multi‐Electron Reactions Enabled by Anion‐Based Redox Chemistry for High‐Energy Multivalent Rechargeable Batteries,” Angewandte Chemie International Edition 59 (2020): 11483–11490, 10.1002/anie.202002560. 32220137 PMC7384178

[15] S. Kim , L. Yin , M. H. Lee , et al., “High‐Voltage Phosphate Cathodes for Rechargeable Ca‐Ion Batteries,” ACS Energy Letters 5 (2020): 3203–3211, 10.1021/acsenergylett.0c01663.

[16] C. Lee , Y. Jeong , P. Maldonado , H. Song , and Y. Kim , “Electrochemical Intercalation of Ca^2+^ ions Into TiS_2_ in Organic Electrolytes at Room Temperature,” Electrochemistry Communications 98 (2019): 115, 10.1016/j.elecom.2018.12.003.

[17] M. Hayashi , H. Arai , H. Ohtsuka , and Y. Sakurai , “Electrochemical Insertion/Extraction of Calcium Ions Using Crystalline Vanadium Oxide,” Electrochemical and solid‐state letters 7 (2004): A119–A121.

[18] M. Cabello , F. Nacimiento , R. Alcántara , P. Lavela , C. P. Vicente , and J. L. Tirado , “Applicability of Molybdite as an Electrode Material in Calcium Batteries: A Structural Study of Layer‐type Ca_x_MoO_3_ ,” Chemistry of Materials 30 (2018): 5853–5861.

[19] M. E. A. Dompablo , C. Krich , J. Nava‐Avendaño , N. Biškup , M. R. Palacín , and F. Barde , “A Joint Computational and Experimental Evaluation of CaMn_2_O_4_ Polymorphs as Cathode Materials for Ca Ion Batteries,” Chemistry of Materials 28 (2016): 6886–6893.

[20] A. L. Lipson , B. Pan , S. H. Lapidus , C. Liao , J. T. Vaughey , and B. J. Ingram , “Rechargeable Ca‐Ion Batteries: A New Energy Storage System,” Chemistry of Materials 27 (2015): 8442–8447, 10.1021/acs.chemmater.5b04027.

[21] B. Ji , H. He , W. Yao , and Y. Tang , “Recent Advances and Perspectives on Calcium‐Ion Storage: Key Materials and Devices,” Advanced Materials 33 (2021): 2005501. 10.1002/adma.202005501 33251702

[22] D. Larcher and J. M. Tarascon , “Towards Greener and More Sustainable Batteries for Electrical Energy Storage,” Nature chemistry 7 (2015): 19–29, 10.1038/nchem.2085. 25515886

[23] J. Xie and Q. Zhang , “Recent Progress in Multivalent Metal (Mg, Zn, Ca, and Al) and Metal‐Ion Rechargeable Batteries With Organic Materials As Promising Electrodes,” Small 15 (2019): 1805061. 10.1002/smll.201805061 30848095

[24] K. Zhao , X. Wang , W. Feng , et al., “Carbonyl‐Based Organic Electrode Materials Spanning From Nonaqueous Rechargeable Lithium to Calcium Batteries,” Energy & Environmental Science 18 (2025): 7756–7791, 10.1039/D4EE06017B.

[25] M. S. Chae , A. Nimkar , N. Shpigel , Y. Gofer , and D. Aurbach , “High Performance Aqueous and Nonaqueous Ca‐Ion Cathodes Based on Fused‐Ring Aromatic Carbonyl Compounds,” ACS Energy Letters 6 (2021): 2659–2665, 10.1021/acsenergylett.1c01010.

[26] D. Monti , N. Patil , A. P. Black , et al., “Polyimides as Promising Cathodes for Metal–Organic Batteries: A Comparison Between Divalent (Ca^2+^, Mg^2+^) and Monovalent (Li^+^, Na^+^) Cations,” ACS Appl Energy Materials 6 (2023): 7250–7257, 10.1021/acsaem.3c00969. PMC10336839 37448980

[27] Y. Ma , Q. Qi , Q. Meng , et al., “A Small Molecular Cathode for High‐Performance Calcium Metal Batteries,” Advanced Functional Materials 35 (2025): 2411715, 10.1002/adfm.202411715.

[28] R. Bitenc , A. Scafuri , K. Pirnat , et al., “Electrochemical Performance and Mechanism of Calcium Metal‐Organic Battery,” Batteries and Supercaps 4 (2021): 214–220.

[29] O. Lužanin , J. Moškon , T. Pavčnik , R. Dominko , and J. Bitenc , “Unveiling True Limits of Electrochemical Performance of Organic Cathodes in Multivalent Batteries Through Cyclable Symmetric Cells,” Batteries and Supercaps 6 (2023): 202200437, 10.1002/batt.202200437.

[30] O. Lužanin , A. K. Lautar , T. Pavčnik , and J. Bitenc , “Paving the Way for Future Ca Metal Batteries Through Comprehensive Electrochemical Testing of Organic Polymer Cathodes,” Materials Advances 5 (2023): 642–651.

[31] X. Guo , R. Markowski , A. Black , et al., “Amorphous Coordination Polymers for Versatile Mg^2+^, Ca^2+^, Sr^2+^, Ba^2+^, and Zn^2+^ Cation Storage,” Energy & environmental science 18 (2025): 9114–9124, 10.1039/D5EE02567B. 40969188 PMC12441785

[32] S. Zhang , Y. Zhu , S. Ren , et al., “Covalent Organic Framework with Multiple Redox Active Sites for High‐Performance Aqueous Calcium Ion Batteries,” Journal of the American Chemical Society 145 (2023): 17309–17320, 10.1021/jacs.3c04657. 37525440

[33] A. Jouhara , N. Dupré , A.‐C. Gaillot , D. Guyomard , F. Dolhem , and P. Poizot , “Raising the Redox Potential in Carboxyphenolate‐Based Positive Organic Materials Via Cation Substitution,” Nature communications 9 (2018): 4401, 10.1038/s41467-018-06708-x. PMC6199296 30353001

[34] A. Ponrouch , J. Bitenc , R. Dominko , N. Lindahl , P. Johansson , and M. R. Palacin , “Multivalent Rechargeable Batteries,” Energy Storage Materials 20 (2019): 253–262, 10.1016/j.ensm.2019.04.012.

[35] J. Forero‐Saboya , C. Davoisne , R. Dedryvère , I. Yousef , P. Canepa , and A. Ponrouch , “Understanding the Nature of the Passivation Layer Enabling Reversible Calcium Plating,” Energy & Environmental Science 13 (2020): 3423–3431, 10.1039/D0EE02347G.

[36] X. Liu , G. A. Elia , and S. Passerini , “Evaluation of Counter and Reference Electrodes for the Investigation of Ca Battery Materials,” Journal of Power Sources Advances 2 (2020): 100008, 10.1016/j.powera.2020.100008.

[37] S. Bai , B. Kim , C. Kim , et al., “Permselective Metal‐Organic Framework gel Membrane Enables Long‐Life Cycling Of Rechargeable Organic Batteries,” Nat Nanotechnology 16 (2021): 77–84, 10.1038/s41565-020-00788-x. 33139935

[38] Y. Wang , X. Song , S. Liu , G. Li , S. Ye , and X. Gao , “Elucidating the Effect of the Dopant Ionic Radius on the Structure and Electrochemical Performance of Ni‐Rich Layered Oxides for Lithium‐Ion Batteries,” ACS applied materials & interfaces 13 (2021): 56233–56241, 10.1021/acsami.1c17991. 34787405

[39] C. Ribeiro , R. Markowski , R. F. Mendes , et al., “Structure–Performance Relationships in Anthraquinone‐Disulfonate Coordination Polymers for Li‐Ion and Na‐Ion Battery Cathodes,” Batteries & Supercap 8 (2025): 202500360.

[40] Y. Tao , Y. Cui , H. Wang , Z. Li , Z. Qian , and P. Zhang , “High‐Efficiency Electrochemical Desalination: The Role of a Rigid Pseudocapacitive Polymer Electrode With Diverse Active Sites,” Advanced Functional Materials 35 (2025): 2414805, 10.1002/adfm.202414805.

[41] J. Yang , Y. Tao , C. Zhao , Y. Cai , P. Xiao , and M. Shi , “Tailorable Dual‐Redox Polymer With Molecular Flexibility for Enhanced Electrochemical Desalination and Water Purification,” Environmental Science & Technology 59 (2025): 10980–10989, 10.1021/acs.est.5c04042. 40454462

[42] X. Lin , P. Apostol , H. Xu , et al., “Design Principles of Quinone Redox Systems for Advanced Sulfide Solid‐State Organic Lithium Metal Batteries,” Advanced Materials 36 (2024): 2312908, 10.1002/adma.202312908. 38843480

[43] S. Renault , S. Gottis , A.‐L. Barrès , et al., “A Green Li–Organic Battery Working as a Fuel Cell in Case of Emergency,” Energy & Environmental Science 6 (2013): 2124–2133, 10.1039/c3ee40878g.

[44] S. Zhang , Y. Zhu , D. Wang , et al., “Poly (anthraquinonyl Sulfide)/CNT Composites as High‐Rate‐Performance Cathodes for Nonaqueous Rechargeable Calcium‐Ion Batteries,” Advanced Science 9 (2022): 2200397, 10.1002/advs.202200397. 35306763 PMC9108664

[45] K. Wakamatsu , S. Ohkata , M. Kajiwara , N. Tanifuji , H. Yoshikawa , and T. Shimizu , “Ultrafast High‐Capacity Calcium‐Ion Battery Based on Efficient Calciation Into the Two‐Dimensional Structure of a Cu3(2,3,6,7,10,11‐hexahydroxytriphenylene)_2_ Cathode,” ACS Electrochemistry 2 (2026): 596–603.

[46] N. Kuperman , P. Padigi , G. Goncher , D. Evans , J. Thiebes , and R. Solanki , “High Performance Prussian Blue Cathode for Nonaqueous Ca‐Ion Intercalation Battery,” Journal of Power Source 342 (2017): 414–418, 10.1016/j.jpowsour.2016.12.074.

