# Supplementary material for: Carboxyphenolate Coordination Frameworks for High‐Voltage Calcium Storage
Source: Adv Sci (Weinh). 2026 May 11;13(43):e75481. doi: 10.1002/advs.75481 (PMC13335451; doi:10.1002/advs.75481)
Supplement: Supplementary file 1 — Supporting File: advs75481‐sup‐0001‐SuppMat.docx. [file ADVS-13-e75481-s001.docx]

*Supporting Information for*

Carboxyphenolate Coordination Frameworks for High-Voltage Calcium Storage

Vasudeva Rao Bakuru, ^a*^ Darsi Rambabu, ^a^ Xiaodong Lin, ^a^ Robert Markowski, ^a^ Petru Apostol, Viliam Frano, ^a^ Xiaolong Guo, ^a^ Taniya Purkait, ^a^ Shubhadeep Pal, ^a,b^ Tom Goossens, ^a^ Da Tie, ^a^ Augustin Ramackers,^a^ and Alexandru Vlad ^a,c*^

^a^ Institute of Condensed Matter and Nanosciences, Molecular Chemistry, Materials and Catalysis, Université catholique de Louvain, Louvain-la-Neuve B-1348, Belgium

^b^ Department of Physics, School of Advanced Science, VIT-AP University, Amaravati-522241, Andhra Pradesh, India.

^c^  WEL Research Institute, avenue Pasteur, 6, 1300 Wavre, Belgium

*Email: [vasudeva.bakuru@uclouvain.be](mailto:vasudeva.bakuru@uclouvain.be), [alexandru.vlad@uclouvain.be](mailto:alexandru.vlad@uclouvain.be)

Contents

*1. Synthesis of Ca_2_-M-THBPD (M: Mg^2+^, Ca^2+^ and Ba^2+^) 2*

*2. Characterization 3*

*3. Solubility check in electrolytes 7*

*4. Electrochemical performances 8*

*5. Chemic oxidation of Ca_2_-M-THBPD using NOBF_4_ 21*

*6. Reference 22*

1. Synthesis of Ca_2_-***M***-THBPD (M: Mg^2+^, Ca^2+^ and Ba^2+^)

*Synthesis of Ca_2_-****M-****THBPD (M:Mg*^2+^ *or Ba*^2+^*):* The H_6_-THBPD ligand was synthesized following our previous report^[1]^. In a typical procedure, H_6_-THBPD (100 mg, 0.32 mmol, 1.0 equiv.) and anhydrous sodium methoxide (28.86 mg, 1.95 mmol, 6.2 equiv.) were dissolved in anhydrous methanol (10 mL) inside an argon-filled glovebox and stirred for 24 h. Subsequently, the corresponding metal salt was introduced into the solution: Mg(TFSI)_2_ (1.0 equiv.) for Ca_2_**-*Mg*-**THBPD, or BaCl_2_ (1.0 equiv.) for Ca_2_**-*Ba*-**THBPD. The mixtures were further stirred for 24 h. For the Ca_2_**-*Mg*-**THBPD and Ca_2_**-*Ba*-**THBPD samples, an additional 2.2 equivalents of CaCl_2_ in methanol were then added, and the reaction was allowed to proceed for another 24 h. The resulting solids were collected by centrifugation, washed several times with methanol and diethyl ether, and dried under vacuum at 120 °C for 2 h, 150 °C for 6 h, and 220 °C for 10 h using a Büchi oven to afford the corresponding Ca_2_**-*M*-**THBPD frameworks.

*Synthesis of Ca_2_-****Ca-****THBPD:* Following a similar procedure, H_6_-THBPD (100 mg, 0.32 mmol) and sodium methoxide (28.9 mg, 1.95 mmol) were dissolved in anhydrous methanol (10 mL) and stirred for 24 h. CaCl_2_ (113.5 mg, 3.2 equiv.) in methanol was then added, and the mixture was stirred for 48 h. The solid product was collected, washed with methanol and diethyl ether, and dried under the same vacuum heating protocol to afford Ca_2_**-*Ca*-**THBPD as a powder.

2. Characterization

**Figure S1.** Structural and morphological characterization of Ca_2_-***M***-THBPD materials: A) XRD patterns (top), B) and C) N_2_ adsorption-desorption isotherms (77 K) with BJH pore size distributions (middle), and D) SEM images showing distinct morphologies dense aggregates for Ca_2_-***Mg***-THBPD, E) open porous aggregates for Ca_2_-***Ca***-THBPD, and F) hierarchical, interconnected porous aggregates for Ca_2_-***Ba***-THBPD (bottom).

X-ray diffraction (XRD) patterns of Ca_2_-***M***-THBPD (M^2+^ = Mg^2+^, Ca^2+^, Ba^2+^) exhibit flat, featureless profiles without distinct Bragg peaks, confirming their amorphous nature which arises from kinetically controlled metal-ligand coordination under strongly basic conditions with excess sodium methoxide, sequential cation addition, the flexibility of the THBPD ligand, and the incorporation of different alkaline-earth metals; this amorphous structure is advantageous for Ca-ion batteries as it enhances Ca^2+^ mobility, accommodates volume changes, and mitigates diffusion limitations typical of crystalline hosts. As these systems exhibit low periodic order, determining their structure is highly challenging. We propose that the carboxylate groups at either end of the ligand facilitate the formation of extended oligo- or polymeric coordination complexes, which may result in amorphous coordination frameworks.

The specific surface areas were determined by N_2_ adsorption-desorption at 77 K (**Figure S1B, C**). The isotherms display type II behavior, indicative of non-porous or macroporous structures with textural adsorption.The Brunauer-Emmett Teller (BET) surface areas of Ca_2_-***Mg***-THBPD, Ca_2_-***Ca***-THBPD, and Ca_2_-***Ba***-THBPD were 167, 96, and 46 m^2^ g^-1^, respectively, reflecting relatively low surface areas typical of compact coordination frameworks lacking permanent microporosity.

SEM characterization has been performed for all three materials (**Figure S1D-F**). The SEM images reveal distinct morphologies (agglomerated spherical particles) for the three samples with particle sizes of approximately 100 nm. Ca_2_-***Mg***-THBPD consists of densely packed aggregates with a rough, compact structure. Ca_2_-***Ca***-THBPD exhibits more loosely connected aggregates with an open, porous structure, while Ca_2_-***Ba***-THBPD displays hierarchical, heterogeneous aggregates forming an interconnected, porous network. These morphological features are consistent throughout the series and align with the amorphous character inferred from the absence of Bragg reflections in the PXRD patterns.


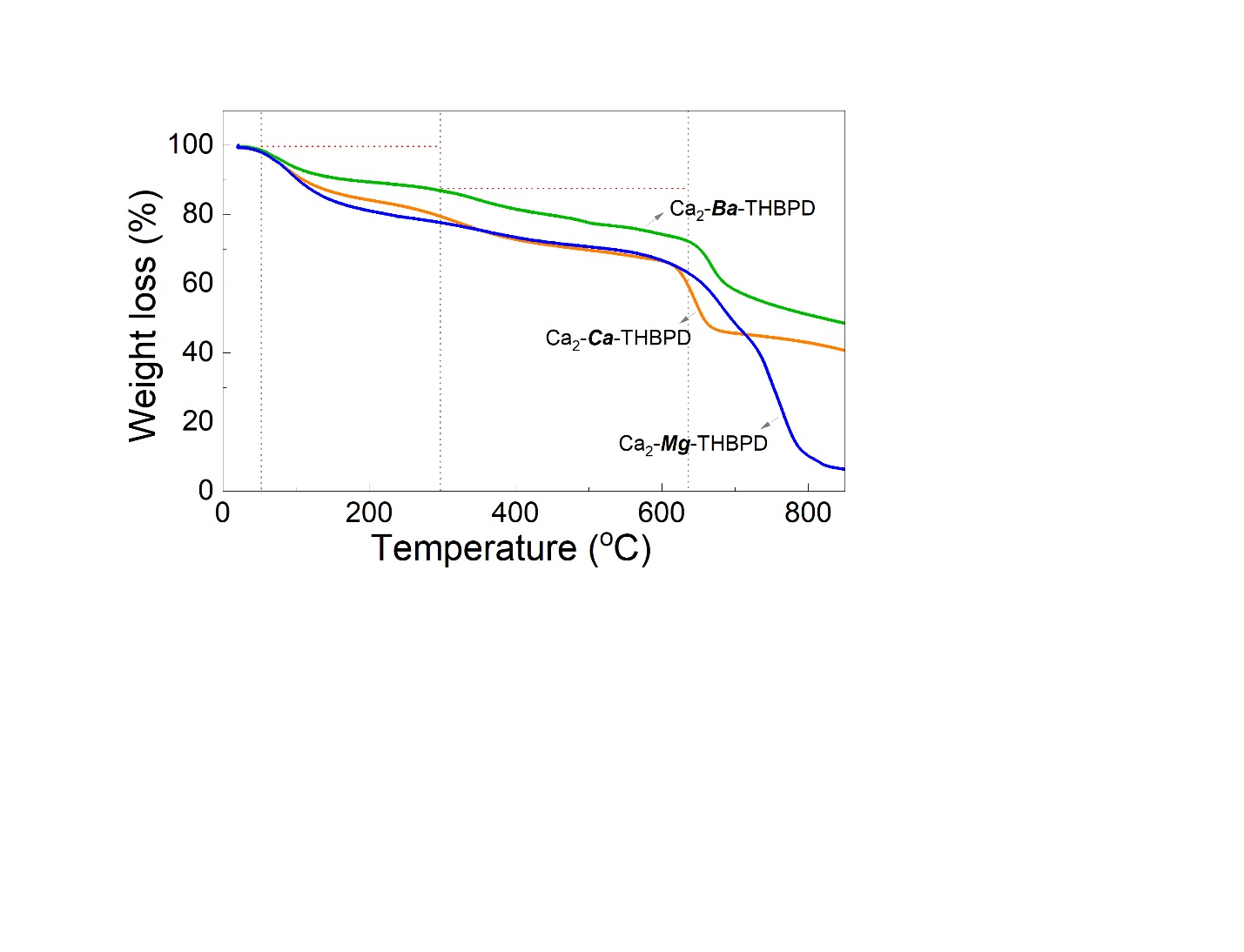


**Figure S2:** Thermogravimetry analysis (TGA) of Ca_2_-***M***-THBPD positive electrode materials. Conditions: Analysis was carried out under N_2_, from room temperature to 850˚C with ramp rate of 10˚C /min.

A representative thermogravimetric analysis (TGA) profile of three Ca_2_-***M***-THBPD (M^2+^ = Mg^2+^, Ca^2+^, Ba^2+^) positive electrode materials reveals an initial weight loss between 50 and 120 °C, which can be attributed to the desorption of adsorbed water from the atmosphere during sample transfer prior to measurement. The Ca_2_-***Mg***-THBPD sample shows the highest weight loss (~23 wt%), compared to Ca_2_-***Ca***-THBPD (~20 wt%) and Ca_2_-***Ba***-THBPD (~13 wt%). Following this first decomposition step, all samples exhibit enhanced thermal stability beyond ~300 °C, indicating the complete removal of residual solvents and moisture.

**Table S1**. Elemental analysis (CH and ICP-OES) of Ca_2_-***M***-THBPD positive electrode materials.

| **Compound** | **Weight %** | **Carbon**  **(C)** | **Hydrogen**  **(H)** | **Magnesium**  **(Mg)** | **Calcium**  **(Ca)** | **Barium**  **(Ba)** | **Sodium**  **(Na)** |
| --- | --- | --- | --- | --- | --- | --- | --- |
| Ca_2_-***Mg***-THBPD | Theor. | 41.56 | 1.00 | 6.01 | 19.52 | - | - |
|  | Exp. | 40.61 | 1.87 | 5.79 | 18.71 | - | 0.65 |
| Ca_2_-***Ca***-THBPD | Theor. | 40.00 | 0.96 | - | 28.19 | - | - |
|  | Exp. | 40.40 | 1.53 | - | 29.05 | - | - |
| Ca_2_-***Ba***-THBPD | Theor. | 32.48 | 0.78 | - | 15.48 | 26.53 | - |
|  | Exp. | 35.99 | 1.42 | - | 16.19 | 17.11 | 0.24 |

3. Solubility check in electrolytes

The solubility of Ca_2_-***Ca***-THBPD was further examined in 1 M Ca(TFSI)_2_ solutions prepared in acetonitrile and propylene carbonate (PC). As shown in the image below, the material remained insoluble in both electrolyte systems, indicating its robust stability under typical electrochemical conditions.


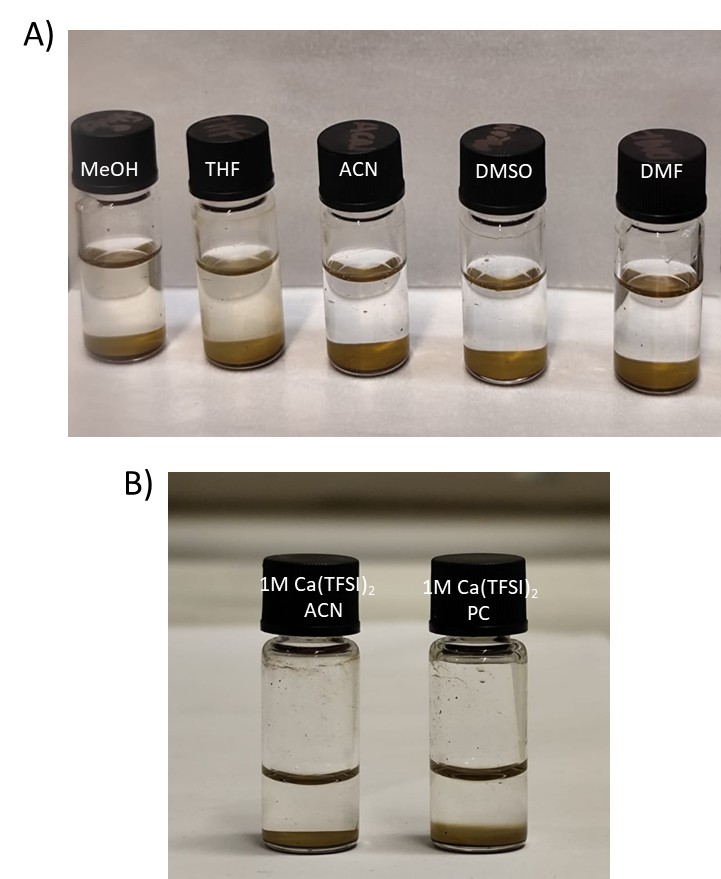


**Figure S3**. Solubility assessment of Ca_2_-***Ca***-THBPD electrode. Insolubility in 1 M Ca(TFSI)_2_ electrolytes prepared in acetonitrile and propylene carbonate (PC).

4. Electrochemical performances

*Assembly and testing of three-electrode cells:* The assembly of the three-electrode cells was executed within an argon (Ar)-filled glovebox to mitigate the influence of atmospheric contaminants. Employing active carbon and a silver (Ag) wire as the counter and pseudo-reference electrodes, respectively, the evaluation of working potential (versus Ag metal) for Ca_2_-***Ca***-THBPD positive electrodes was conducted through galvanostatic charge-discharge method using a VMP galvanostat/potentiostat (manufactured by Bio-Logic, France). Subsequently, the potential of the Ag wire pseudo-reference electrode was calibrated via cyclic voltammetry at a scan rate of 100 mV s^-1^. This calibration procedure was carried out within a three-electrode cell configuration comprising a glassy carbon electrode (GCE) as the working electrode, a platinum electrode as the counter electrode, and an Ag metal electrode serving as the pseudo-reference electrode. The electrolytic medium consisted of a 1M solution of calcium bis(trifluoromethanesulfonyl)imide (Ca (TFSI)_2_) in acetonitrile solvent, supplemented with 0.01 M ferrocene as an internal reference compound.

From **Figures S4A**, we can deduce: φ _Ca2-_***_Ca_***_-THBPD_ = φ_Ag_^+^_/Ag_ + 0.35V

From **Figure S4C**: φ_Ag_^+^_/Ag_ = φ_Fc_^+^_/Fc_ **-** 0.15V

Given that: φ_Fc_^+^_/Fc_ = φ_Ca_^2+^_/Ca_ + 3.29V

We can derive the following relationships: φ_Ca2-_***_Ca_***_-THBPD_ = φ_Ca_^2+^_/Ca_ + 3.5V

**Figure S4.** A) Three-electrode galvanostatic charge-discharge profile at C/20, with Ca_2_-***Ca***-THBPD as the working electrode (WE), activated carbon as the counter electrode (CE), and a silver wire pseudo-reference (RE). B) Cyclic voltammogram of 10 mM ferrocene in 1 M Ca(TFSI)_2_ in ACN at 100 mV/s, recorded with a glassy carbon working electrode, platinum counter electrode, and silver wire pseudo-reference (φₐ, anodic; φ_c_, cathodic).

As shown in **Figure S5B**, the principal redox peaks exhibit a gradual decrease in intensity with cycling when Super-P is used as the conductive additive, whereas their potential positions remain largely invariant and no pronounced peak splitting or systematic voltage shift is observed. This behavior indicates that the capacity fading is not primarily governed by increasing polarization or kinetic hindrance, which would manifest as significant peak displacement and broadening. Instead, the progressive attenuation of peak intensity is consistent with a reduction in the fraction of electrochemically accessible redox sites, pointing to active material loss or partial electrochemical isolation as the dominant degradation mechanism. Notably, this interpretation is fully consistent with the evolution of the galvanostatic profiles, and the capacity retention trend shown in **Figure S5A**, as well as the relatively stable coulombic efficiency upon cycling **(Figure S5D)**. The multi-cycle dQ/dV analysis therefore provides a mechanistic basis for the observed capacity decay and substantially strengthens the electrochemical discussion. In contrast to Super P, KB600 enhances stabilization **(Figure S5F)**, limits dissolution, and improves conductivity, leading to better utilization. Accordingly, dQ/dV peaks intensify and sharpen upon cycling, indicating increased electrochemical accessibility and redox-site activation.

The solid-state CV profiles (**Figure S5C**) display defined anodic oxidation (0.23V, 0.37V) and cathodic reduction (0.18V, 0.37V) features that are highly reproducible upon cycling. After the initial cycle, the voltammograms show substantial overlap, indicating rapid establishment of a stable electrochemical regime and a high degree of reversibility of the redox processes. Importantly, the separation between the oxidation and reduction peaks remains limited and does not increase with cycle number, demonstrating low polarization and favorable charge-transfer kinetics. This behavior is further corroborated by the corresponding dQ/dV plots (2nd cycle: anodic oxidation (0.14V, 0.4V) and cathodic reduction (0.14V, 0.4V), which clearly resolve the redox events and confirm the persistence and stability of the electrochemical signatures over extended cycling. The absence of significant peak shifting or broadening with increasing cycle number rules out progressive kinetic limitations or structural degradation. Overall, the CV analysis provides direct experimental evidence for reversible redox behavior and stable reaction kinetics of the electrode material, thereby reinforcing the conclusions drawn from the galvanostatic measurements.

**Figure S5**: Electrochemical performance of Ca_2_-***Ca***-THBPD: (A) Galvanostatic charge-discharge profiles at selected cycles (1^st^, 2^nd^, 3^rd^, 5^th^, and 10^th^) at a constant current rate of 25 mA/g (corresponding to a rate of C/10). (B) Corresponding dQ/dV curves. (C) Solid state CV of Ca_2_-***Ca***-THBPD electrode material at current rate of 0.05 mV s^-1^. (D) Cycling stability and Coulombic efficiency. Composite: Active material (50%), Super-p (40%) and PTFE binder (5%). Electrolyte: 0.5 M acetonitrile in Ca(TFSI)_2_. E) UV-Vis spectra of chemically oxidized Ca_0_-***Ca***-THBPD and the electrode after full charge. F) Corresponding dQ/dV curves. Composite: Active material (50%), Ketjen black (40%) and PTFE binder (5%). Electrolyte: 1 M acetonitrile in Ca(TFSI)_2_.

**Figure S6.** Scanning electron microscopy (SEM) images of (A) pristine Ca_2_-**Ca**-THBPD, (B) Super-P conductive carbon, and (C) composite before electrochemical testing. (D-E) SEM images of the composite electrode after prolonged cycling. (F) Photographs of the separators collected after 20 cycles.

SEM image of pristine Ca_2_-***Ca***-THBPD shows an aggregated particulate morphology composed of irregularly shaped grains (**Figure S6A**). Super-P displays typical nanoscale carbon aggregates with smaller particle size (50 nm) (**Figure S6B**) where as Ca_2_-***Ca***-THBPD particles are comparatively larger (~100 nm). In the composite electrode, Ca_2_-***Ca***-THBPD particles are homogeneously distributed within the Super-P matrix. The Ca_2_-***Ca***-THBPD particles (highlighted by yellow dashed circles shown in **Figure S6C**), remain well embedded within the conductive carbon network, which forms a continuous percolating pathway to ensure efficient electronic connectivity. After cycling (**Figure S6D-E**), the electrode largely retains its overall morphology; however, localized surface changes are observed, including slight smoothing and the formation of more rounded features in selected regions. These modifications be associated with interfacial reorganization during repeated redox cycling. While limited dissolution of the oxidized phase into the electrolyte cannot be excluded, the evidence remains indirect. This is further supported by the photograph of the separators collected after 20 cycles (**Figure S6F**), which shows slight discoloration.

We have performed a comprehensive GITT study at a C/10 rate, applying 1 h current pulses followed by 5 h relaxation periods to allow the system to reach quasi-equilibrium. The resulting voltage profiles display well-defined steps corresponding to sequential Ca^2+^ insertion and extraction events. From these data, the diffusion coefficient (D) was calculated using following equation (1) for both charge and discharge processes. The results reveal variations in D across the cycle, reflecting the influence of electrode structure and ion mobility. Averaging D over the full cycle provides a representative value of 2.95 × 10^-10^ cm^2^/s, which quantitatively characterizes Ca^2+^ transport within the electrode. In addition, for first discharge, the diffusion coefficient from 4.1 × 10^-10^ (*D at high-potential) to 1.0 × 10^-10^ cm^2^ s^-1^ (D at low-potential) indicating relatively fast multivalent ion transport in the solid-state electrode. These GITT-derived diffusion coefficients therefore provide a robust assessment of Ca^2+^ kinetics, complementing the electrochemical performance data presented.

The diffusion coefficient D was calculated using:

$D=\frac{4}{\pi\tau}$(m_B_V_m_/M_B_S)^2^(ΔE_s_/ΔE_τ_)^2^  - (1)

Here, τ denotes the duration of the galvanostatic charge/discharge pulse. $m_{B}$, $M_{B}$, and $V_{m}$correspond to the active material mass, molar mass, and molar volume, respectively, while S represents the geometric surface area of the electrode. ΔE_s_ is the change in open-circuit voltage (OCV) between two consecutive current pulses, and ΔE_τ_ is the voltage variation during the applied current pulse. GITT measurements were performed using a Neware battery cycler, and the diffusion coefficients were calculated using the Neware-BTSDA 8.0 software after inputting the relevant parameters. The molar volume (V_m_) was estimated using a density of 1.03 g cm^-3^, determined from pellet measurements based on the calculated thickness and electrode area.

**Figure S7.** A) The Galvanostatic Intermittent Titration Technique (GITT) profiles of the electrode at C/10 (1 h pulses, 5 h rest) showing voltage steps during ion extraction/ insertion, and B) Diffusion coefficients (D) during full cycle. The overall average D over the full cycle (1st cycle) is 2.95 × 10^-10^ cm^2^/s. *Composite*: Ca_2_-**Ca**-THBPD: Supper-P: PTFE (5:4:1).

In situ Electrochemical Impedance Spectroscopy (EIS acquired after each electrochemical step, which consisted of a 3 h charge or discharge period followed by a 1 h relaxation period at a current density of 35 mA g^-1^) measurements were carried out using 2025-type coin cells with active carbon fabric as the counter/reference electrode on a Bio-Logic VSP-300 potentiostat. The Nyquist plots (**Figure S8A**) display similar high-frequency intercepts for all states, confirming comparable ohmic resistances, in agreement with the Bode analysis. A depressed semicircle in the mid-frequency region (**Figure S8B**) indicates charge-transfer processes at the electrode/electrolyte interface, with slight variations depending on the applied potential, consistent with differences observed in the phase angle maxima in the Bode plots. At low frequencies, the emergence of an inclined line is characteristic of Warburg-type behavior, reflecting diffusion-limited processes.^[3][4]^ This is further supported by phase angles (**Figure S8C& D**) approaching 45° in the Bode plots, indicating that the electrochemical response is primarily governed by the sluggish diffusion of Ca^2+^ ions within the framework. Together, these results highlight that while the overall electrochemical mechanism remains unchanged, the kinetics are modulated by structure-dependent variations in ion transport and interfacial charge-transfer processes.

**Figure S8.** Kinetic analysis of the Ca_2_-***Ca***-THBPD positive electrode. A) In situ Nyquist plots, Bode plots collected at various charged and discharged states; B) Magnitude plot: Shows the impedance magnitude |Z| (in ohms) versus frequency, C) Phase angle plot: Shows the phase angle φ (in degrees) versus frequency. D) The corresponding contour map derived from Bode plots recorded at different charge and discharge states. EIS spectrum was acquired after each electrochemical step, which consisted of a 3 h charge or discharge period followed by a 1 h relaxation period at a current density of 35 mA g^-1^.


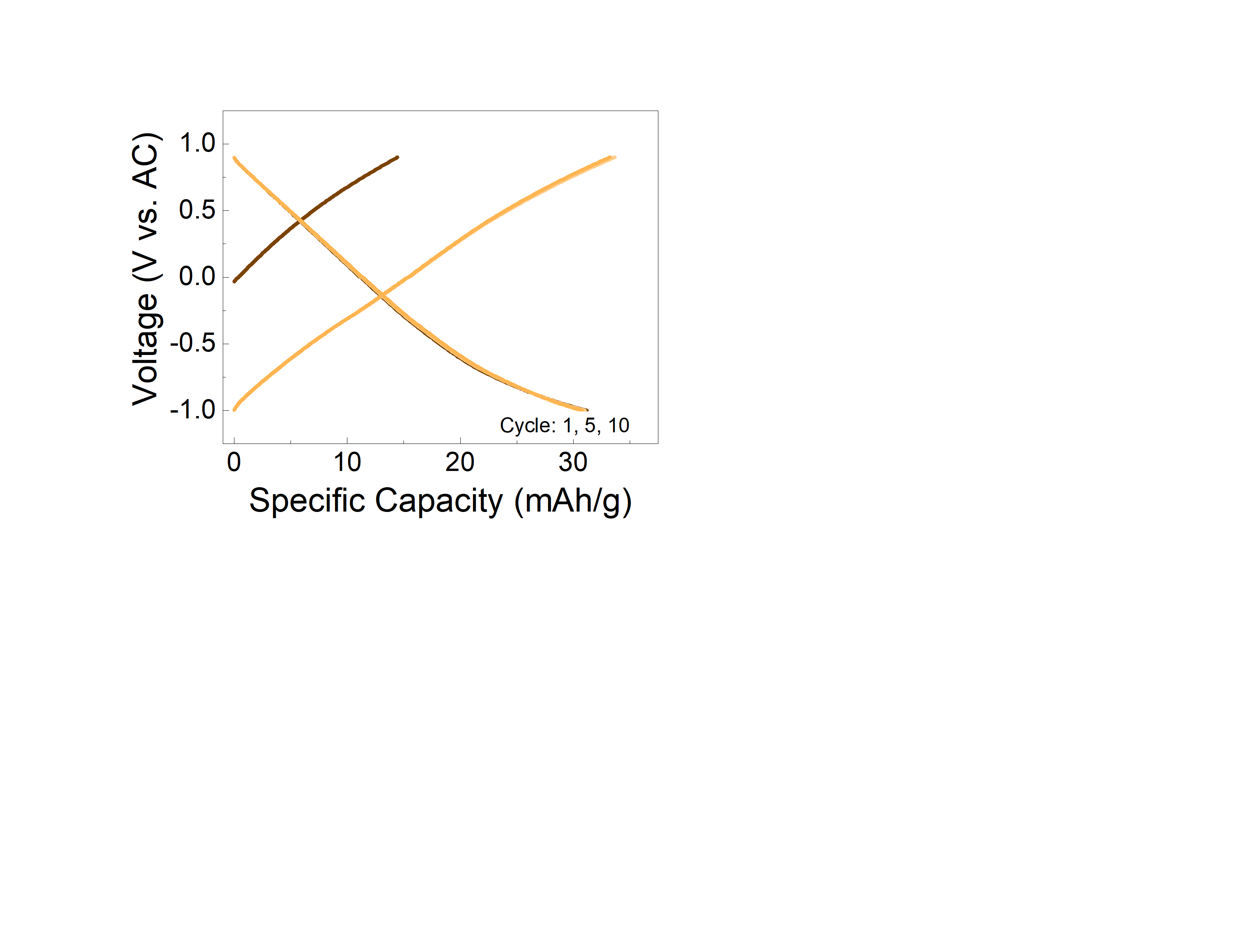


**Figure S9.** Galvanostatic charge-discharge curves of Ketjen Black (KB) vs. activated carbon (AC) showing symmetric capacitive behavior with 32 mAh g^-1^ reversible capacity.


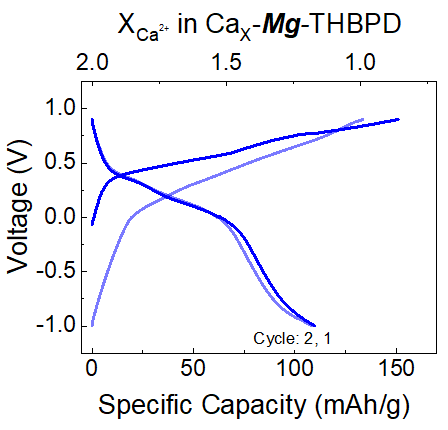


**Figure S10**. A) Galvanostatic charge-discharge profiles of the Ca_2_-***Mg***-THBPD positive electrode material over the first two cycles at a rate of C/20, measured between -1.0 and 0.9 V vs. activated carbon in 1 M Ca(TFSI)_2_/acetonitrile electrolyte.


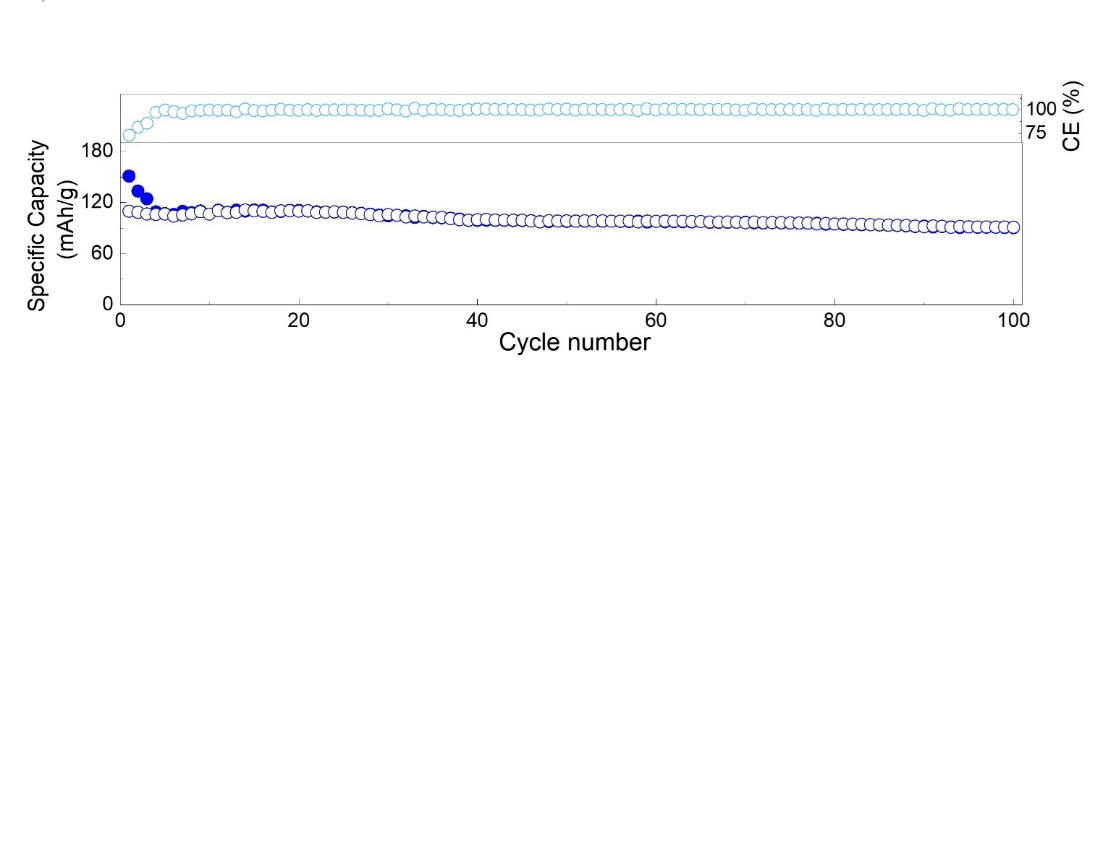


**Figure S11**. Cycling performance of the Ca_2_-***Mg***-THBPD electrode at a constant current rate of C/20.


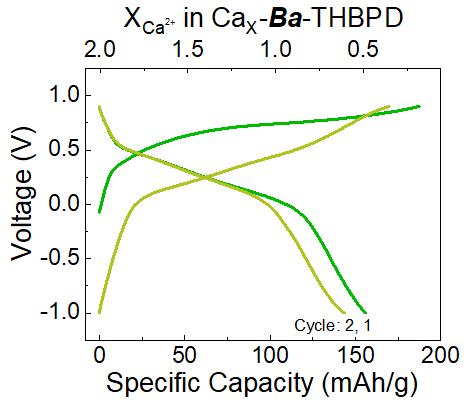


**Figure S1**2. A) Galvanostatic charge-discharge profiles of the Ca_2_-***Ba***-THBPD positive electrode material over the first two cycles at a rate of C/20, measured between -1.0 and 0.9 V vs. activated carbon in 1 M Ca(TFSI)_2_/acetonitrile electrolyte.


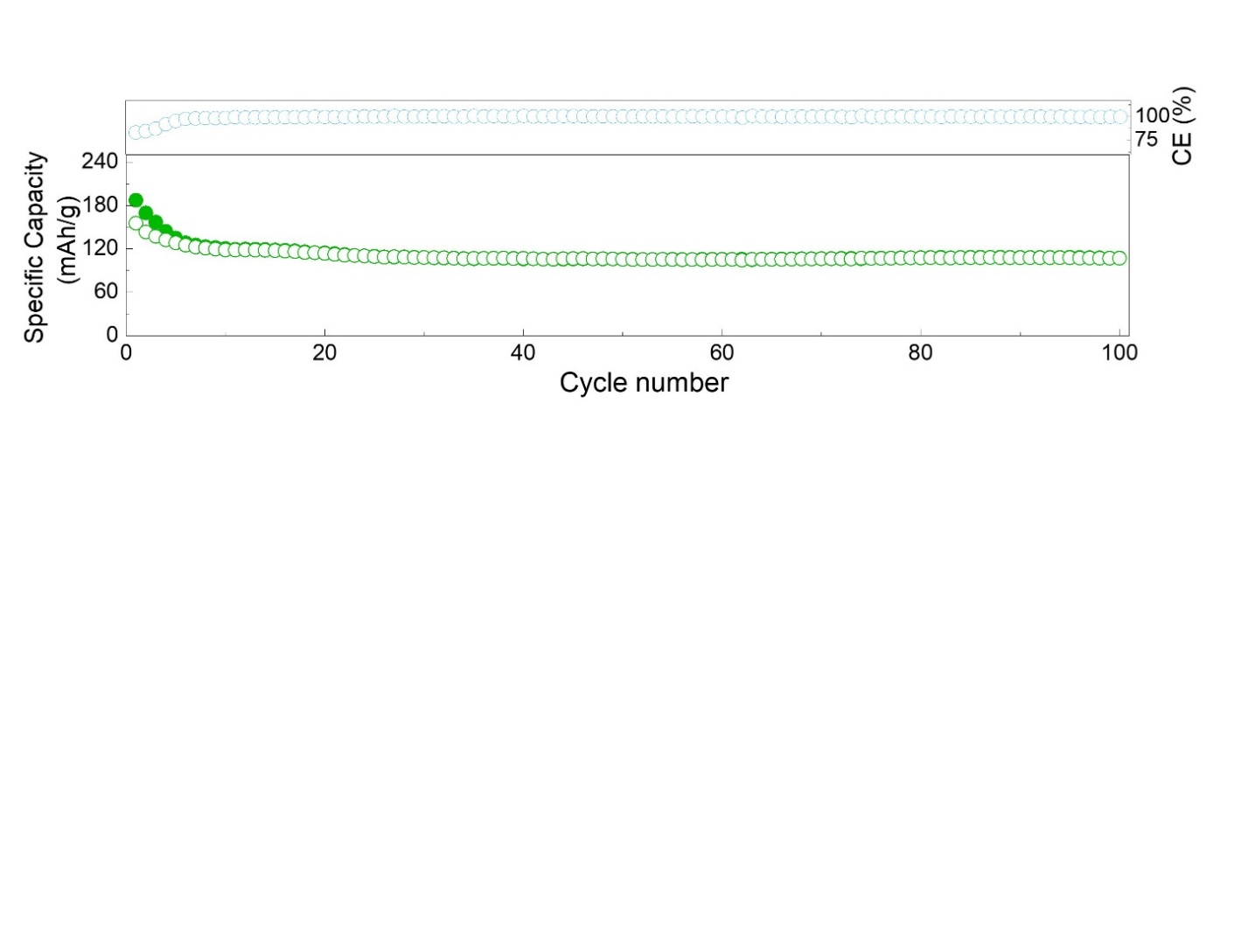


**Figure S13**: Cycling performance of the Ca_2_-***Ba***-THBPD electrode at a constant current rate of C/20.

**Table S2.** Comparison of Ca_2_-***M***-THBPD with reported inorganic and organic Ca-ion positive electrodes in terms of capacity, average voltage, and energy density.

| Materials | Electrolyte | | Energy density  (Wh kg^-1^/C-rate) | | Capacity  /mAh g^-1^ | | Average discharge voltage/ V | | Ref. | |
| --- | --- | --- | --- | --- | --- | --- | --- | --- | --- | --- |
| *Inorganic positive electrode materials* | | | | | | | | | | |
| K_0.5_V_2_O_5_ | 0.5 M Ca(ClO_4_)_2_ PC | | 280 (0.1C) | | 100 | | 2.8 | | ^[5]^ | |
|  |  |  | 243 (0.3C) | | 87 | |  |  |  |  |
|  |  |  | 196 (0.5C) | | 70 | |  |  |  |  |
|  |  |  | 140 (1C) | | 50 | |  |  |  |  |
| Ca_x_Na_0.5_VPO_4.8_F_0.7_ | 1.0 M Ca(PF_6_)_2_EC:PC | | 288 (0.08C) | | 87 | | 3.2 | | ^[6]^ | |
|  |  |  | 265 (0.16C) | | 83 | |  |  |  |  |
|  |  |  | 233 (0.4C) | | 74 | |  |  |  |  |
|  |  |  | 198 (0.8C) | | 62 | |  |  |  |  |
|  |  |  | 169 (1.6C) | | 54 | |  |  |  |  |
|  |  |  | 137 (4C) | | 43 | |  |  |  |  |
| NaV_2_(PO_4_)_3_ | 0.5 M Ca(TFSI)_2_ DME | | 259 (0.04C) | | 80 | | 3.2 | | ^[7]^ | |
| Ag_0.33_V_2_O_5_ | 0.5 M Ca(BF_4_)_2_ in EC/PC | | 336 (0.2C) | | 120 | | 2.8 | | ^[8]^ | |
|  |  |  | 252 (0.5C) | | 90 | |  |  |  |  |
|  |  |  | 84 (1C) | | 30 | |  |  |  |  |
| α-MnO_2_ | 0.5 M Ca(TFSI)_2_ in ACN) | | 184 (0.33C) | | 60 | |  | | ^[9]^ | |
| β-MnO_2_ |  |  | 87.4(0.33C) | | 29 | | 3.02 | |  |  |
| γ-MnO_2_ |  |  | 145 (0.33C) | | 48 | |  | |  |  |
| VS_4_ | 0.25 M Ca[B(hfip)_4_]_2_/DME | | 630 (0.3C) | | 315 | | 2.0 | | ^[10]^ |  |
| CaV_6_O_16_ 2.8H_2_O | 0.3 M Ca(TFSI)_2_  /G_2_ | | 330 (0.2C) | | 106 | | 3.1 | | ^[11]^ | |
|  |  |  | 270(0.4C) | | 87 | |  |  |  |  |
|  |  |  | 200 (0.8C) | | 64 | |  |  |  |  |
|  |  |  | 120 (2C) | | 38 | |  |  |  |  |
|  |  |  | 90 (3.2C) | | 29 | |  |  |  |  |
|  |  |  | 80 (6.4C) | | 25 | |  |  |  |  |
| NiFe(CN)_6_ | 0.2 M Ca(PF_6_)_2_ EC:PC | | 150 (0.05C) | |  | | 2.6 | | ^[12]^ |  |
| Materials | Electrolyte | Energy density  /Wh kg^-1^/C-rate | | Capacity  /mAh g^-1^ | | Avg. discharge voltage  / V | | Ref. | | |
| *Organic positive electrode material* | | | | | | | | | | |
| PQ | 2.5 M Ca(TFSI)_2_ TEGDME:DOL | 685(0.2C) | | 250.0 | | 2.74 | | ^[13]^ | | |
|  |  | 599(0.3C) | | 218.8 | |  |  |  |  |  |
|  |  | 489(0.4C) | | 178.8 | |  |  |  |  |  |
|  |  | 405(0.5C) | | 148.1 | |  |  |  |  |  |
| PAQs@CNTs | 0.8 M Ca(TFSI)_2_ EC:DMC:PC:EMC | 348 (0.43C) | | 116 | | 3.0 | | ^[14]^ | | |
|  |  | 318(0.94C) | | 106 | |  |  |  |  |  |
|  |  | 303(1.98C) | | 101 | |  |  |  |  |  |
|  |  | 246(12.19C) | | 82 | |  |  |  |  |  |
|  | Ca(ClO_4_)_2_ in PC |  | | | | | | | | |
|  |  | 318 (0.16C) | | 138 | | 2.3 | |  | | |
|  |  | 316 (0.32C) | | 137 | |  |  |  | | |
| PTCDA |  | 308 (0.64C) | | 133 | |  |  | ^[15]^ | | |
|  |  | 280 (1.28C) | | 121 | |  |  |  | | |
|  |  | 244 (2.5C) | | 106 | |  |  |  | | |
|  | 0.5 M Ca(TFSI)_2_ in EC:PC | 178 (0.05C) | | 100 | |  | | ^[16]^ | | |
|  |  | 174 (0.1C) | | 98 | |  | |  |  |  |
| PNTCDA |  | 168 (0.5C) | | 87 | | 2.0 | |  |  |  |
|  |  | 163 (1C) | | 80 | |  | |  |  |  |
| Ca-Co-PTtSA | Ca (TFSI)_2_ DME | 342 (0.07C | | 106 | | 3.22 | | ^[17]^ | | |
|  |  | 326(0.1C) | | 101 | |  |  |  |  |  |
|  |  | 313(0.2C) | | 97 | |  |  |  |  |  |
|  |  | 289(0.5C) | | 89 | |  |  |  |  |  |
|  |  | 262 (1C) | | 81 | |  |  |  |  |  |
|  |  | 304 (0.1C) | | 94 | | 3.24 | |  |  |  |
|  |  | 291 (0.2C) | | 90 | |  |  |  |  |  |
| Ca-Zn-PTtSA |  | 270 (0.5C) | | 83 | |  |  |  |  |  |
|  |  | 158 (1C) | | 48 | |  |  |  |  |  |
|  |  | 244 (2C) | | 75 | |  |  |  |  |  |
|  |  | 214 (5C) | | 66 | |  |  |  |  |  |
|  |  | 172 (10C) | | 53 | |  |  |  |  |  |
| Ca_2_-***Mg***-THBPD | 1M Ca(TFSI)_2_ ACN/ PC | 315(0.05C) | | 90 | | 3.5 | | This work | | |
| Ca_2_-***Ca***-THBPD |  | 420(0.05C) | | 120 | |  |  |  |  |  |
| Ca_2_-***Ba***-THBPD |  | 420(0.05C) | | 120 | |  |  |  |  |  |

In addition to the above reports, a TB-COF has been extensively studied for Ca-ion storage in aqueous media.^[18]^ However, both Ca^2+^ and H^+^ contribute to the overall capacity. Carbonyl (C=O) groups are emphasized because they provide strong, well-defined coordination with Ca^2+^, enabling stable and reversible redox behavior. Other functionalities, such as C=N groups, may also participate via O/N coordination, but their interaction strength and redox activity are generally weaker and less reliable, making carbonyl-based motifs the most established platforms for Ca^2+^ storage.

5. Chemical oxidation of Ca_2_-***Ca***-THBPD using nitrosonium tetrafluoroborate (NOBF_4_)


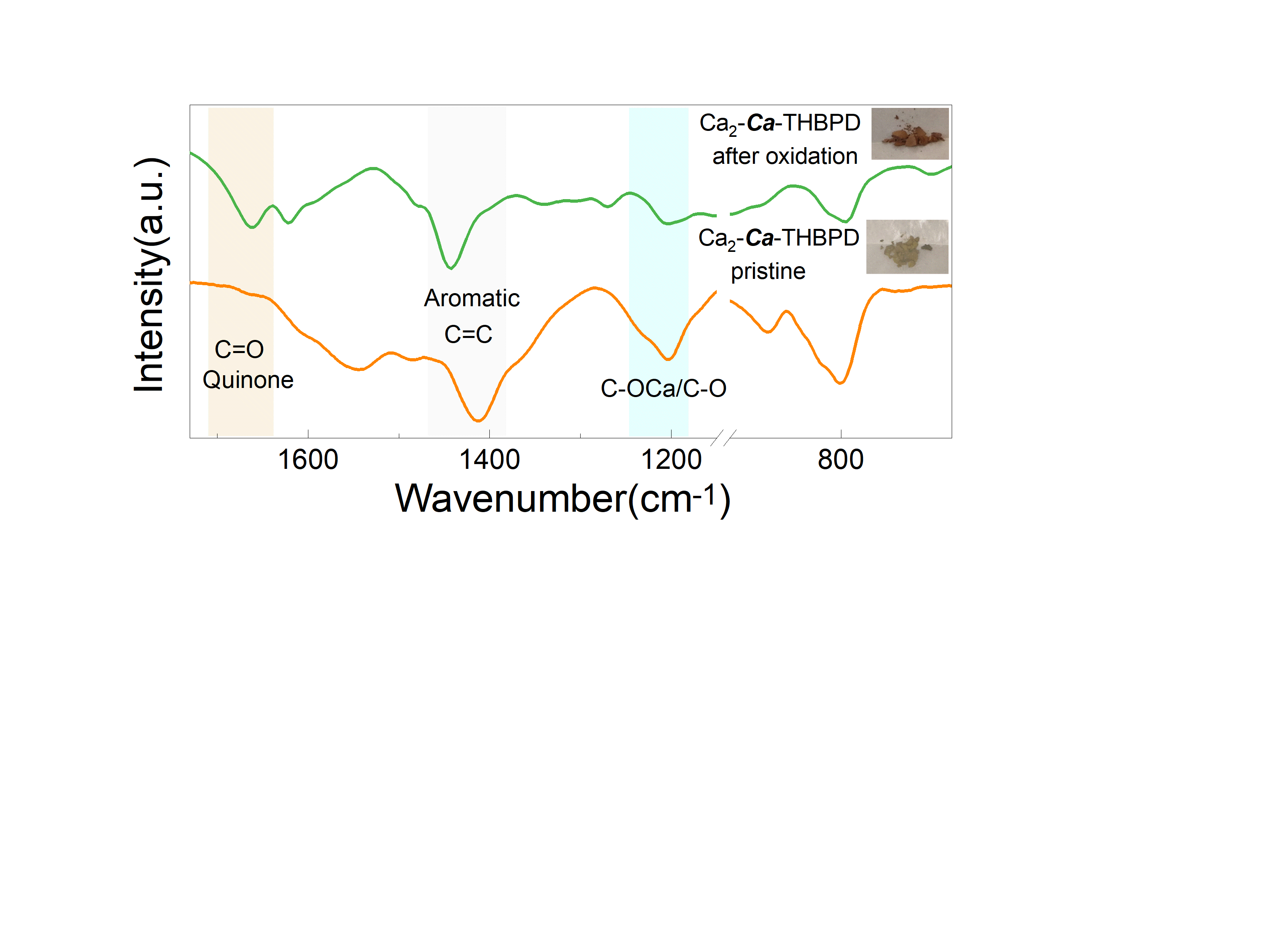


**Figure S14.** Chemical oxidation analysis of Ca_2_-***Ca***-THBPD product. Associated FTIR spectra before and after chemical oxidation of Ca_2_-***Ca***-THBPD.

Typically, 0.1mmol of Ca_2_-***Ca***-THBPD (green in color) was dispersed in acetonitrile, and 0.4 mmol (4 equivalents for 4e^-^) of NOBF_4_ were added, and the resulting reaction mixture stirred for 24 hours at room temperature under inert atmosphere. After the reaction, the mixture was separated washed with acetonitrile and diethyl ether. Finally, the products (dark brown in color) were dried at 150 °C, and the samples were characterized as described next.

The oxidized compounds were characterized by Fourier-transform infrared (FTIR) spectroscopy (**Figures S14**). In the fully oxidized Ca_2_-***Ca***-THBPD positive electrode materials, the emergence of a distinct carbonyl stretching vibration (ν_C=O_) at 1661 cm^-1^ is observed, indicative of the formation of quinone functionalities. Additionally, the shift of the aromatic C=C stretching band from 1413 to 1443 cm^-1^ upon oxidation indicates decreased π-electron density and increased bond stiffness, consistent with electron withdrawal from the aromatic ring and a ligand-centered oxidation process.

6. References

[1] V. R. Bakuru, P. Apostol, D. Rambabu, S. Pal, X. Lin, R. Markowski, T. Goossens, D. Tie, A. Kachmar, Y. Zhang, G. Chanteux, A. Vlad, *Energy Environ. Sci.* **2025**, *18*, 6131.

[2] S. Bai, B. Kim, C. Kim, O. Tamwattana, H. Park, J. Kim, D. Lee, K. Kang, *Nat. Nanotechnol.* **2021**, *16*, 77.

[3] J. Yang, Y. Tao, C. Zhao, Y. Cai, P. Xiao, M. Shi, *Environ. Sci. Technol. 2025,* **2025**, *59*, 10980−10989.

[4] Y. Tao, Y. Cui, H. Wang, Z. Li, Z. Qian, P. Zhang, *Adv. Funct. Mater. 2025,* **2025**, *2414805*, 1.

[5] M. E. Purbarani, J. Hyoung, S. T. Hong, *ACS Appl. Energy Mater.* **2021**, *4*, 7487.

[6] Z. L. Xu, J. Park, J. Wang, H. Moon, G. Yoon, J. Lim, Y. J. Ko, S. P. Cho, S. Y. Lee, K. Kang, *Nat. Commun.* **2021**, *12*, 1.

[7] S. Kim, L. Yin, M. H. Lee, P. Parajuli, L. Blanc, T. T. Fister, H. Park, B. J. Kwon, B. J. Ingram, P. Zapol, R. F. Klie, K. Kang, L. F. Nazar, S. H. Lapidus, J. T. Vaughey, *ACS Energy Lett.* **2020**, *5*, 3203.

[8] J. Hyoung, J. W. Heo, B. Jeon, S. T. Hong, *J. Mater. Chem. A* **2021**, *9*, 20776.

[9] C. Zuo, F. Xiong, J. Wang, Y. An, L. Zhang, Q. An, *Adv. Funct. Mater.* **2022**, *32*, 2202975.

[10] Z. Li, B. P. Vinayan, P. Jankowski, C. Njel, A. Roy, T. Vegge, J. Maibach, J. M. G. Lastra, M. Fichtner, Z. Zhao‐Karger, *Angew. Chemie Int. Ed.* **2020**, *59*, 11483.

[11] J. Wang, J. Wang, Y. Jiang, F. Xiong, S. Tan, F. Qiao, J. Chen, Q. An, L. Mai, *Adv. Funct. Mater.* **2022**, *32*, 2113030.

[12] A. L. Lipson, S. D. Han, S. Kim, B. Pan, N. Sa, C. Liao, T. T. Fister, A. K. Burrell, J. T. Vaughey, B. J. Ingram, *J. Power Sources* **2016**, *325*, 646.

[13] Y. Ma, Q. Qi, Q. Meng, Y. Yi, H. Lin, J. Yu, C. F. Cheung, Z. Xu, *Adv. Funct. Mater.* **2025**, *35*, 2411715.

[14] S. Zhang, Y. Zhu, D. Wang, C. Li, Y. Han, Z. Shi, S. Feng, *Adv. Sci.* **2022**, *9*, 2200397.

[15] M. S. Chae, A. Nimkar, N. Shpigel, Y. Gofer, D. Aurbach, *ACS Energy Lett.* **2021**, *6*, 2659.

[16] D. Monti, N. Patil, A. P. Black, D. Raptis, A. Mavrandonakis, G. E. Froudakis, I. Yousef, N. Goujon, D. Mecerreyes, R. Marcilla, A. Ponrouch, *ACS Appl. Energy Mater.* **2023**, *6*, 7250.

[17] X. Guo, R. Markowski, A. Black, P. Apostol, D. Rambabu, O. Lužanin, T. Pavčnik, D. Monti, M. Du, D. Tie, X. Lin, V. R. Bakuru, R. Delogne, K. Robeyns, L. Simonelli, J.-F. Gohy, J. Bitenc, J. Wang, A. Ponrouch, A. Vlad, *Energy Environ. Sci.* **2025**, *18*, 9114.

[18] S. Zhang, Y. Zhu, S. Ren, C. Li, X. Chen, Z. Li, Y. Han, Z. Shi, S. Feng, *J Am Chem Soc.* **2023**, 17309−17320.
